# Carcass and Meat Quality Traits of Males and Females of the “*Branca*” Portuguese Autochthonous Chicken Breed

**DOI:** 10.3390/ani12192640

**Published:** 2022-09-30

**Authors:** Márcio Meira, Isabel M. Afonso, Susana Casal, Júlio Cesar Lopes, Jéssica Domingues, Virgínia Ribeiro, Rui Dantas, José V. Leite, Nuno V. Brito

**Affiliations:** 1 Escola Superior Agrária, Instituto Politécnico de Viana do Castelo, Rua Escola Industrial e Comercial de Nun’Álvares, 4900-347 Viana do Castelo, Portugal; 2 CISAS, Escola Superior Agrária, Instituto Politécnico de Viana do Castelo, Rua Escola Industrial e Comercial de Nun’Álvares, 4900-347 Viana do Castelo, Portugal; 3 Requimte—LAQV, Laboratório de Bromatoologia e Hidrologia, Faculdade de Farmácia, Universidade do Porto, Rua de Jorge Viterbo Ferreira, 228, 4050-313 Porto, Portugal; 4 AMIBA—Associação dos Criadores de Bovinos de Raça Barrosã, 4730-260 Vila Verde, Portugal; 5 TOXRUN—Toxicology Research Unit, University Institute of Health Sciences, CESPU, CRL, 4585-116 Gandra, Portugal

**Keywords:** poultry, local breeds, carcass yield, mineral composition, fatty acids

## Abstract

**Simple Summary:**

Local breeds play a crucial role in the rural economies of many countries, as a considerable valuable genetic resource and, well adapted to the environment, their production can reduce the negative impact of intensive production systems. Portuguese chicken breeds are almost extinct, with the “Branca” population presenting the most worrying situation. Bred as a dual-purpose breed, the characterization of meat quality is fundamental to the conservation and promotion of this population., by increasing the value on these small-scale farms production. The carcass characteristics and meat quality of the “Branca” breed were evaluated, showing an interesting physicochemical profile, with good proportion of minerals, essential fatty acids (EFA) and n-3-PUFAs (docosapentanoic (C22:5n-3, DPA), and docosahexaenoic (C22:6n-3, DHA) acids), ensuring that consumers receive a highly nutritional and differentiated product.

**Abstract:**

The “Branca” breed is a dual-purpose Portuguese autochthonous chicken breed, produced in extensive systems and in small flocks, especially in the Entre Douro and Minho regions. A total of 40 birds (n = 20/sex) were slaughtered between 38 and 42 weeks (males) and 110 and 120 weeks (females), and carcass and meat quality parameters were evaluated. The results showed significantly higher weights and differences for males between sexes and pieces (*p* ≤ 0.05) in the meat physicochemical composition. Water holding capacity (WRC), moisture and ash contents were influenced by sex. They were higher in males in the breast and drumstick and lightness (L*) and lipid content were higher in females (*p* ≤ 0.05). The breast meat presented greater lightness, moisture, ash and protein contents, while the drumstick showed a higher pH value, redness (a*) and lipid content, in both sexes (*p* ≤ 0.05). Regarding the nutritional properties, “Branca” meat revealed a good proportion of minerals and the female meat showed, in both pieces (*p* ≤ 0.05), significantly lower values for total saturated fatty acids (SFAs) and higher values for monounsaturated fatty acids (MUFAs). Breast meat was richer in n-3-PUFAs (C22:5n-3 and C22:6n-3) in both sexes (*p* ≤ 0.05). Considering the results obtained, it can be concluded that “Branca” breed meat is a healthy food characterized by a good general nutritional profile.

## 1. Introduction

The use of chicken hybrids with high-performing productivity in intensive poultry farming constitutes a serious risk to the adaptation to global warming, to emerging diseases and to complex changes in consumer demand [1,2]. Nowadays, the sustainable use of local breeds in extensive systems is presented as an alternative practice to industrial farming.

According to the report of the Commission on Genetic Resources for Food and Agriculture (CGRFA), among avian species, chickens are the ones with the greatest number of breeds at risk on a global scale [3,4]. It is estimated that 103 of the 1.640 chicken breeds identified worldwide have become extinct, of which, 95 were in Europe and the Caucasus [4].

In Portugal, all of the four autochthonous chicken breeds: “Amarela”, “Branca”, “Pedrês Portuguesa” and “Preta Lusitânica”, are classified as endangered, although the “Branca” is the one that presents the most worrying situation due to the fact that it was the last to be recognized [5,6]. Produced in free-range conditions, with a simple, functional and traditional construction and adapted to the number of animals and type of production (meat or eggs), females are used to produce eggs, while males are kept for meat production and commonly sold as a whole carcass [5,7,8].

More than animal genetic resources, local breeds have significant sociocultural and ecological relevance, can improve local communities economy and reduce the negative impact of intensive farming systems [8,9]. The use of autochthonous populations to satisfy the demands of consumers should be encouraged to help safeguard biodiversity, enhancing the diffusion of native chicken breeds by ensuring their use in the production of alternative poultry products [9].

Given the initiatives and changes in consumption habits, today the demand for higher quality poultry products has increased significantly [10,11]. This demand has progressively focused on product quality traits, animal welfare and consumer concerns about health and nutritional diet quality [1,9].

The nutritional quality of poultry meat depends on the content of high-value protein, unsaturated fatty acids, vitamins, macro and micronutrients and other biologically active compounds [12,13]. Although native breeds have a lower growth performance than commercial lines, from a nutritional viewpoint their meat is more attractive and healthier (more protein, less fat and higher n-3 PUFA content), ensuring the demand of particular consumer segments [10,11,12,13].

**Figure 1:**
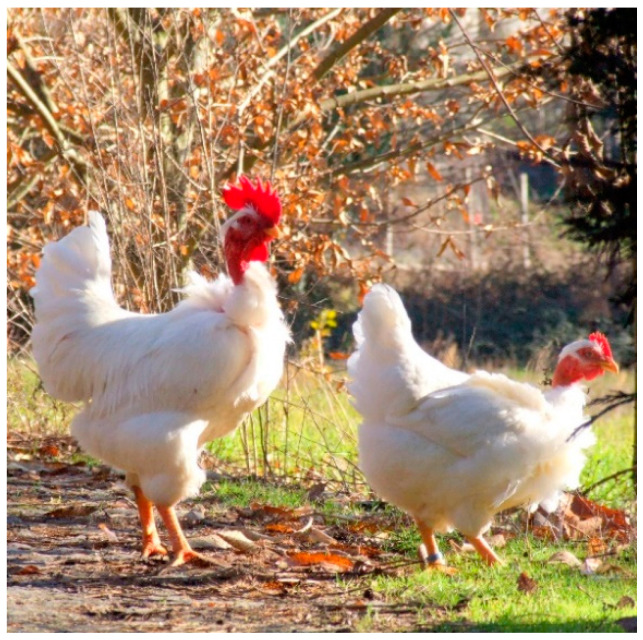
Portuguese autochthonous Branca breed (male and female).

Considering the scarce amount of scientific data concerning the meat quality of the Portuguese poultry breeds, the present study aims to describe the meat quality parameters of males and females of the “Branca” breed (Figure 1), in order to support their conservation, increase their intrinsic value and correspond to the new trends of consumer demand.

## 2. Materials and Methods

The study was carried out at the Agrarian Higher School of the Polytechnic Institute of Viana do Castelo (ESA-IPVC). All the procedures described were approved by the organization responsible for the Animal Welfare of the Polytechnic Institute of Viana do Castelo (ORBEA-IPVC), in accordance with the Decree-Law No. 113/2013, of August 7.

### 2.1. Sample Size and Animal Management

The sample consisted of 40 birds, 20 males and 20 females, originating from AMIBA (Associação dos Criadores de Bovinos de Raça Barrosã) and associated breeders; the animals were identified as being 21 days of age, through an identifier (earring), placed in the anterior crease of the right-wing. On this earring was engraved, on one side, the serial number referring to the animal and, on the other side, three letters associated with the breed.

All animals were raised in free-range conditions (in the same geographical area), on small family farms, using a simple, functional and traditional construction adapted to the number of animals and type of production (meat or eggs), with a maximum indoor density of 4 to 6 birds/m^2^, and a minimum outdoor area of 4 m^2^/animal.

During the first 21 days of life, the birds were fed with a standard commercial starter and water ad libitum. Then, they had free access to pasture and were fed ad libitum with corn or a mixture of cereals and, when available, were provided with other vegetables surpluses or by-products from the farms, such as cabbages, carrot, pumpkin and lettuce among others.

### 2.2. Slaughtering and Carcass Traits Evaluations

Following traditional methods of production and consumption, the animals were slaughtered at different ages: males between 38 and 42 weeks, and females between 110 and 120 weeks, ages at which, generally, they are replaced and consumed.

For the carcass traits evaluation, the selected animals were weighed (live weight (LW)) and slaughtered after fasting for 12 to 16 h. The birds were mechanically stunned, killed by manual exsanguination (carotid and jugular incision) during approximately 30 to 40 s, manually plucked after scalding, washed and then weighed (weight of plucked and bled carcass (CW1)). After evisceration and cooling (24 h at 4 °C) two other weight measurements were performed, corresponding to the eviscerated carcass weight with (CW2) or without head, paws and edible viscera (CW3).

Another weight (EW) was estimated due to the importance of popular consumption traditions that include the carcass (CW2) plus all edible viscera (head, paws, gizzard, heart, liver and kidneys). Corresponding carcass yields were obtained by expressing those weights as percentages of LW (CY1, CY2, CY3 and EY, respectively). In females, CW2, EW and their yields do not include the head.

### 2.3. Analytical Determinations 

The physicochemical parameters of the 40 breast and 40 drumstick pieces were analysed. Meat samples were prepared in the laboratory, consisting of the removal of the skin and bones, which were cut and homogenized for 2 min (Grindomix GM200, Retsch, Haan, Germany), and then, divided into two parts. One portion was used to determine pH, moisture, ash, protein and lipid contents, according to the AOAC (2000) [14], and the remaining part was freeze-dried and analysed for the fatty acid profile and mineral composition.

Colour determination was measured at 24 h post-mortem, using a portable colorimeter Chroma Meter CR-400 Minolta (Konica Minolta, Osaka, Japan) calibrated with a white standard plate (Y = 84.7, x = 0.3173, y = 0.3237), with an 8 mm diameter measuring area, C illuminant and 2° standard observer. The results were expressed in terms of lightness (L*), redness (a*) and yellowness (b*) in the CIELAB colour space (Commission Internationale de l’Éclairage) [15]. Colour values were obtained considering the average of five readings per sample on the inner surface of the breast and drumstick muscle. The pH of the samples was measured at 24-h post mortem using a digital portable pH-meter (HALLO^TM^ FC2022, Hanna Instruments, Eibar, Spain) equipped with a penetration probe, previously calibrated using standardized buffers (pH 4.0 and 7.0), according to the ISO 2917:1974 method [16].

The water-holding capacity (WHC) of the breast and drumstick muscle was estimated by loss of water by centrifugal force [17]; approximately 1 g of the muscle, placed on filter paper inside a tube, was centrifuged for 4 min at 1500× *g*. The water remaining after centrifugation was quantified by drying the samples at 70 °C overnight. The result expressed as a percentage of water retained by the sample was obtained by the difference (weight after centrifugation − weight after drying)/initial weight × 100.

The weight difference before and after applying pressure was used to determine the loss of water (%) in the breast and drumstick muscle [18]; approximately 5 g of muscle was submitted to a weight of 2250 g between two filter papers for 5 min. The result expressed as a percentage of water lost by the sample was obtained by the difference in weight before and after the method was performed (initial weight − final weight)/initial weight × 100.

Moisture, following the drying method up to constant weight at 105 °C in a stove, was quantified according to the ISO recommended standard 1442:1997 [19]. Ash content was estimated with incineration (muffle B150, Nabertherm, Germany) of the samples for 6 h and a temperature of 550 °C ± 25 °C, according to ISO 936:1978 method [20]. 

Protein content was determined according to the Kjeldahl method (ISO 937:1978), with the digestion (DK 20, Heating Digestor, Velp Scientifica), distillation (UDK 139, semi-automatic distillation V. Scientifica) and titration (Titroline 5000, SI Analytics) of samples, multiplying the amount of azote by a factor of 6.25 [21].

Total fat content was quantified following the Soxhlet method (behr ED, behr Labor-Technik GmbH, Düsseldorf, Germany) with ether petroleum solvent. The fatty acid profile was determined according to the procedure described by Cruz et al. [22], after extraction of the fat with nonhalogenated organic solvents, followed by methylation with 0.5 M potassium hydroxide and boron trifluoride, in methanol, and separation in a CP-Select FAME chromatographic column (100 m, Agilent, Santa Clara, CA, USA), with adequate calibration standards.

The contents of the main (phosphorous (P), potassium (K), calcium (Ca), magnesium (Mg), sodium (Na)) and trace (iron (Fe), zinc (Zn), manganese (Mn) and copper (Cu)) minerals were determined according to the procedure described by Vale et al. [23]. After digestion (DK 42, Heating Digestor, Velp Scientifica), K, Ca, Mg, Na, Fe, Zn, Mn and Cu were determined in the digested solutions by flame-atomic absorption spectrometry (Perkin Elmer AAnalyst 200, Waltham, MA, USA), while phosphorous content was determined according to the Ascorbic Acid Standard Method [24] in a UV/VIS spectrophotometer (Thermo Scientific Evolution 60S) at 620 nm.

### 2.4. Data Analysis

Descriptive statistics (mean, standard deviation-SD, minimum/maximum values) were generated for all of the variables in the dataset. Analysis of variance was performed on carcass traits evaluations and meat quality data using the IBM SPSS Statistics 23.0 for Windows [25]. The analysis was carried out using a *t*-test of independent samples with a variable grouping of sex and breast and drumstick portions. All statements of significance were based on testing at the *p* ≤ 0.05 level.

## 3. Results

### 3.1. Carcass Traits Evaluations

Carcass characteristics of males and females of the “Branca” breed are shown in Table 1. All carcass parameters, with the exception of CY1, differed between sexes (*p* ≤ 0.05), with males presenting higher LW (3484 vs. 2518 g), higher CW3 (2569 vs. 1683 g) and higher CY3 (73.80 vs. 67.10%), in comparison to females.

### 3.2. Meat Quality Parameters

The physicochemical properties of the breast and drumstick meat of males and females of the “Branca” breed are shown in Table 2 and Table 3, respectively.

Post-mortem pH decline is one of the most important aspects in the conversion of muscle to meat due to its impact on meat texture, colour and water-holding capacity (WHC). No sex significant effect was observed in the pH values in both pieces (Table 2), although, in the drumstick, the pH values were significantly higher (*p* ≤ 0.05) than the breast.

The WHC estimated as percentage of water retained (Table 2) was significantly (*p* ≤ 0.05) affected by sex (male > female) and was more expressive in the breast (55.89 vs. 53.13%). Contrary to WHC, estimated as percentage of water retained, WHC estimated as water loss by pressure (PL) was not influenced by sex (*p >* 0.05), but differed between pieces. The breast muscle showed, in both sexes (*p* ≤ 0.05), a greater loss of water. Concerning colour parameters (Table 2), only lightness (L*) was influenced by sex, with females revealing significantly lighter breast (54.64 vs. 50.12) and drumstick (42.02 vs. 40.39) meat than males (*p* ≤ 0.05). The type of muscle influenced the colour of the meat, with the drumstick showing a lower lightness (L*) value and a high red index (a*) in both sexes (*p* ≤ 0.05).

Chemical composition results (Table 3) showed that sex significantly affected (*p* ≤ 0.05) the moisture and ash contents in the drumstick (females < males), and lipid content in both muscles (males < females). When compared by pieces, breast meat exhibited higher ash, protein and lower lipid contents in relation to the drumstick (*p* ≤ 0.05).

### 3.3. Fatty Acid Profile

The fatty acid (FA) profile is shown in Table 4. Palmitic (C16:0), oleic (C18:1) and linoleic (C18:2n-6) acids were the most representative fatty acids in meat. In relation to the total fractions, monounsaturated fatty acids (MUFAs) were the most abundant, followed by saturated fatty acids (SFAs) and polyunsaturated fatty acids (PUFAs).

Sex proved to be a source of variation between the main FA, with a significant effect on the total profiles of SFAs (males > females) and MUFAs (females > males), as a result of higher expression of stearic acid (C18:0) (*p* ≤ 0.05) in males and C18:1 (*p* ≤ 0.05) in females, respectively.

Although no significant piece effect on the total amounts of SFAs, MUFAs and PUFAs (*p* > 0.05) was observed, the breast was, in both sexes, richer in docosapentanoic acid (C22:5n-3, DPA) and docosahexaenoic acid (C22:6n-3, DHA) (*p* ≤ 0.05) than the drumstick, and thus in n-3-PUFAs (*p* ≤ 0.05).

To assess the nutritional value of “Branca” breed fat, the total essential fatty acids (EFA), PUFAs/SFAs ratio, as well as n-6/n-3, were determined. As observed in Table 4, the proportion of EFA and the PUFAs/SFAs ratio did not differ between the studied effects; however, the n-6/n-3 ratio was significantly different (*p* ≤ 0.05) between pieces, with the breast showing the lowest rates in both sexes.

### 3.4. Mineral Composition

The food mineral composition is an important aspect for human health, and the “Branca” breed meat proved to be a good source of minerals, containing a wide range of macro and trace elements, some of these with important nutritional value, as presented in Table 5.

Similar to physicochemical composition, minerals also differed between the main effects. Male meat showed more K, P, Mg, Na and Zn and less Ca, Fe, Mn and Cu contents compared to females (*p* ≤ 0.05). For both sexes, K, P and Mg contents were significantly higher in the breast (*p* ≤ 0.05), while Na, Fe, Zn and Cu were more relevant in the drumstick (*p* ≤ 0.05).

## 4. Discussion

Recent studies [1,26] investigated the productive performance, slaughter yield and meat quality of different autochthonous breeds, with the objective to characterize their unique meat quality properties and potential in the poultry industry, and showed that these slow-growing chickens can effectively be utilized in alternative production systems.

Concerning the “Branca” breed, the present study results show that carcass traits were significantly influenced by sex, with females showing lower weights and yields than males (i.e., LW, 2518 vs. 3484 g and CY3, 67.10 vs. 73.80%), as a consequence of the sexual dimorphism [27]. Similar weight to that of other European autochthonous populations, raised in similar production systems, were observed, with values ranging between 2425 and 4240 g in the males and 1747 and 2880 g in females [28,29,30,31,32,33].

When comparing CY2 (eviscerated carcass yield, with head, feet and edible viscera) with CY3 (common presentation of commercial chicken on the market), yields decreased 10% in males and 7% in females, approaching commercial chicken values. This fact reduces the product value and profit and supports the traditional system of commercialization. Similar yields were reported by Soares et al., in “Amarela” (81.04%), “Pedrês Portuguesa” (81.11%) and “Preta Lusitânica” (84.79%) breeds (slaughtered at 240 days of age) [32] and Tasoniero et al. in the “Padovana” (81.8%) and “Polverara” (82.9%) breeds (slaughtered at 183 days of age) [26].

The pH values in poultry meat could be influenced by age, rearing management, pre-slaughter practices or intrinsic behaviour typical of indigenous breeds [34]. Lower pH values in females than males (5.78 vs. 5.81) [26,35] and higher pH values in the drumstick (6.06 vs. 6.04, males and females, respectively) were observed [26,29,30,33,35,36], although they were within the normal range for 24-h post-mortem measurements [37,38]. Variations of pH values between pieces are probably explained by the different types of muscles that predominate in the drumstick (oxidative muscles vs. glycolytic muscles in the breast), as the red fibres and their oxidative metabolism possess less glycogen content, thus limiting the muscle pH post-mortem drop [26,39].

The lower WHC observed in the breast (55.89% vs. 53.13%, males and females, respectively), in comparison with the drumstick value (56.74 vs. 54.02%), associated with a greater loss of water, is linked to the pH value of the muscles [38,39,40], because a low pH is often connected to a lower WHC, leading to higher drip and cooking losses [41,42].

Similar results for WHC were reported by Castellini et al. [38] in “Ross” broilers raised in organic system (53.49 vs. 57.45%, breast and drumstick, respectively), slaughtered at 56 or 81 days of age, and Miguel et al. [43] (6.13 vs. 7.81%, breast and drumstick, respectively), for pressing loss in “Castelhana Negra” males (cocks), slaughtered at 29 weeks of age. Results in the same range of values were found in males (roosters) of the “Amarela” and “Pedrês Portuguesa” breeds (15%) [44], males and females of the “Mos” breed (8–13%) [28,30,36,45,46] and the “Padovana” breed (13–14%) [35], although with a different method (cooking loss).

Colour is one of the most important meat quality characteristics [40] especially due to the myoglobin content, which depends on the type of muscle, species and age of the animal [40,47]. Other intrinsic factors, such as muscle and pH, can also influence the meat colour [40]. 

The breast meat of “Branca” females presented greater lightness (L*) (54.68 vs. 50.12) which was lighter than normal (light, L* > 53) relative to males due to the lower pH and WHC [37]. Lighter colour is associated with greater light scattering as a result of greater denaturation of myofibrillar proteins due to a rapid decrease in post-mortem pH changing the reflectance properties of the muscle [48,49] and explaining the differences in the light-scattering properties of sarcoplasmic proteins.

The higher redness (a*) in the drumstick relative to the breast results from the drumstick muscles red fibres, which are rich in myoglobin and haem pigments, in comparison with the white fibres breast, as observed in other autochthonous populations [30,39]. Furthermore, the results of this study demonstrate that muscle pH seems to have influence on the colour of the meat, confirming that higher pH values result in a darker colour, as observed in the drumstick meat [37].

Concerning meat yellowness (b*), diet significantly affects this parameter, due to increased foraging of plant material and corn-based diets (which contain the natural carotenoid pigment xanthophyll) [40]. Similar production and feeding systems justify the present results, which show common colour parameters (L*, a* and b*) of different autochthonous breeds [28,30,36,45].

Meat moisture plays a fundamental role in meat quality, being responsible for its perceived juiciness [50]. Moisture content can be affected by age [51], and a higher content indicates a lower physiological maturity state. With the increase in age, the level of moisture tends to decrease and lipids to increase [40,51], as observed in the female drumstick (*p* ≤ 0,05). In accordance with other authors, the drumstick muscle revealed, in males, more moisture content than the breast muscle [1,28,29,30,34,36,43].

Ash indicates muscle mineral content. These minerals are associated with the organic compounds involved in the muscle contraction process, and its values increase as the animal grows [52], which may explain the highest ash content in males (*p* ≤ 0,05) [1,50,52], as their percentage of muscle tissue is higher than females.

The ash contents verified in our study were similar to those observed with Portuguese autochthonous breeds [32]. Other authors presented slightly higher values, including in native breeds, and differences could be due to the lack of standardization in the productive performances and meat quality traits of the animals [1,28,29,30,36].

Poultry meat is known to be a source of protein of high nutritional quality and easy digestibility [53,54], due to its content in essential amino acids, glutamine, asparagine, lysine, leucine, arginine and alanine [55]. The present study results point to protein values of 24% in the breast and 20% in the drumstick. Protein content was significantly higher (*p* ≤ 0,05) in the breast, and the same tendency was observed in previous studies with autochthonous breeds [26,28,29,30,32,34,36]. This difference has been associated with muscle composition and their capacity for protein deposition [39,56,57]. 

Compared to commercial lines, including studies carried out under organic and conventional conditions, mean values of protein were higher than those reported in improved hybrid commercial breeds for meat production and broilers (19.90–23.53% vs. 16.09–19.42%) [58,59,60], spent hens (21.50–22.37% vs. 10.00–19.46%) [58,61,62] and broilers in an organic system (20.60–22.76% vs. 19.01–19.47%) [38,51,63], breast and drumstick, respectively. These differences can be explained by diverse factors, such as age, feed management and production system [30,35,64,65], with a significant influence on muscle development, which affects the deposition of proteins in meat [11].

Poultry meat is known for being low in fat and the lipid content can be influenced by genotype, sex, age, diet and the production system [38,40,51]. The females revealed a higher lipid content, probably due to the faster tissue growth and fat deposition rate, as observed by different authors [66].

The drumstick presented higher (*p* ≤ 0,05) lipid content, due to its composition, and type of fibres, presenting the red fibres with a high lipid content [30,39,67]. The low lipid content in the breast is a result of the higher proportion of white fibres and their low need for energy storage [1,26].

The reported results are similar to those in males of autochthonous breeds [28,29,43], and lower in spent hens (40, 53 and 80 weeks), with 1.62 to 3.8% in the breast and 3.89 to 21.5% in the thigh, respectively [58,61]. Relative to commercial hybrid breeds, broilers and broilers in an organic system, the drumstick lipid content was lower than the values described in “Cob”, “Ross”, “Kabir”, “Robusta Maculata”, “817C”, “Bresse” and “Rhode Island Red” (2.47–6.04%) [34,38,58,59,63]. Native breeds presented in both pieces, compared to commercial lines, a lower fat content, which may be an important aspect for consumers concerned about fat intake [10,34,68].

The FA profile is influenced by diet, mainly, genotype, age, sex, muscle type and production system [13,35,69,70]. Our results showed C18:1 (MUFA), C16:0 (SFA) and C18:2n-6 (PUFA) as the predominant FA, and represented approximately 60–80% of total FA, as pointed in different studies [1,28,30,33,34,35,36].

As observed in Table 4, the FA profile showed that the predominant FA were MUFAs followed by SFAs and PUFAs. In poultry, the deposition of SFAs and MUFAs in muscle depends, in part, on their presence in the feed and their synthesis in the liver [13,71]. Greater digestion of unsaturated fats reduces the synthesis of SFAs in the liver, though increases in PUFAs content can influence the suppression of MUFAs synthesis by inhibiting the action of the ∆9-desaturase enzyme complex, which is the main enzyme responsible for the conversion of SFAs to MUFAs [71,72]. The influence of sex, with a female MUFA higher fraction, in both pieces, is explained by the higher conversion of C18:0 (SFA) into C18:1 (MUFA), the most representative of MUFAs.

Regarding PUFAs, the results show that the “Branca” breed presented, in both sexes, a good proportion of total and individual PUFAs, mainly EFA C18:2n-6, C20:4n-6 and C18:3n-3 (α-linolenic acid). Given that EFA must be provided as a dietary supplement due to the body’s inability to synthesize it [72,73], the highest levels of C18:2n-6, C20:4n-6 and C18:3n-3 in “Branca” breed meat may be an attractive nutritional quality trait for health-conscious consumers. Due to the importance of PUFAs, in particular n-3, in physiological functions and in their benefits for human health, the presence of C18:3n-3 in food is very important, as it is the main precursor of other n-3-PUFAs. As observed in other autochthonous breeds, the content of C18:3n-3 (0.53–0.66%) was also lower than that of C18:2n-6 (17.05–21.33%), which may be a consequence of the high concentration of C18:2n-6 in corn [30,32,36,45]. As there is competition for desaturase and elongase enzymes during the metabolism of these EFA, excess of C18:2n-6 in the diet can limit the conversion of C18:3n-3 into its long-chain derivates, reducing the ratio of DPA and DHA [74,75]. However, it is possible to verify that breast meat is richer in DPA and DHA and, therefore, in n-3-PUFA.

A low content of SFAs was verified, in both sexes and pieces, and was lower than those described in autochthonous breeds (34.29–43.99%) [28,29,32], and commercial hybrid breeds improved for meat production, broilers, spent hens and broilers in an organic system (33.90–44.47%) [34,38,58,62,63].

The PUFAs/SFAs and n-6/n-3 ratios are widely used to assess the nutritional value of fat [76,77]. A balanced intake of n-6 and n-3 fatty acids is essential to ensure welfare and reduce the incidence of cardiovascular and other chronic diseases [78,79]. According to some nutritional recommendations, the PUFAs/SFAs ratio in the human diet should be greater than 0.45, while the n-6/n-3 ratio should not exceed 4 [78,80]. The nutritional indices show that the PUFAs/SFAs ratio was higher than the minimum recommended value (0.85–0.93), in accordance to other autochthonous breeds (0.64–1.31) [1,28,29,30,34,36].

The values of the n-6/n-3 ratio were higher than the nutritional indices recommended for the human diet, established at 4:1. Eating habits change and there is a current imbalance in the n-6/n-3 ratio observed in human diets, as a result of increased consumption of fat and vegetable oils rich in n-6-PUFA and a decrease in n-3-PUFA rich foods. Current estimates show that the ratio of n-6 to n-3 fatty acids is approximately 10 to 20:1 [30,78,79]. Therefore, in this sense, the present study results are within acceptable values.

The higher n-6/n-3 ratio in the muscle tissues, breast and drumstick in autochthonous populations [28,29,30,32], is explained by the predominance of n-6 fatty acids in corn (≈52%), and the strong relationship between the dietary fat source and tissue content [81]. According to Pateiro et al. a higher proportion of n-3 in the diet is beneficial, resulting in superior nutritional meat indices (a lower SFAs and n-6/n-3 ratio and a higher PUFAs and PUFAs/SFAs ratio) [30].

The mineral meat composition is relevant to the human health, since minerals are necessary for the regulation of cellular function, growth, mechanisms of neuromodulation and other biochemical and physiological functions in the body, and the lack of minerals can cause metabolic disorders, organ damage, chronic diseases and ultimately death [82,83,84]. It is also important to the animal performance and meat quality, contributing to flavour, colour and texture of food [82]. As minerals cannot be synthesized biochemically by living organisms, their deposition in animal muscle depends on factors such as breed, sex, age, diet and water intake, production system, environmental conditions and muscle type [82,85,86].

The results obtained in this study show that K is the predominant macronutrient in “Branca” meat followed by P, while Fe and Zn are the most significant trace elements, similar to the findings reported by Chen et al. in commercial hybrid breeds [58].

Considering that the main sources of K are vegetables, fruits and dairy products, “Branca” breed meat has been proven to be a good source of this macronutrient (≈1800 mg/100 g of meat), contributing in large part to the recommended intake diary (3500 mg/day) [87]. Intake below this value is associated with an increased risk of stroke [88].

Meat is, also, a major source of iron and zinc, and it has been suggested the regular dietary intake of these minor minerals reduces the incidence of many diseases and exerts a beneficial effect on human health. Meat is a good source of Fe because 50–60% is in the heme form, which can be more readily adsorbed [58,83].

## 5. Conclusions

The use of autochthonous populations to satisfy the demands of consumers should be encouraged to safeguard biodiversity, increase their diffusion and ensure their use in the production of alternative poultry products.

Although sex presented a significant effect on carcass characteristics, the results showed that the “Branca” breed had live weight and carcass yields comparable to other European autochthonous chicken breeds raised in similar production systems. Considering nutritional properties, the Branca breed meat revealed a good proportion of minerals, EFA and n-3-PUFAs (DPA and DHA), which makes the product attractive to the consumers and local gastronomy. 

The breast meat presented greater lightness, moisture, ash, and protein contents, while the drumstick showed a higher pH value, redness (a*), and lipid content, both in males and females. A lower SFAs content was observed in breast and drumstick meat. 

From the consumer’s point of view, knowledge of the physicochemical composition of meat and its fatty acid profile can be an important factor for the valorisation the product. The high content of protein and minerals of high biological value associated with a low-fat content (composed mostly of unsaturated fatty acids) with favourable nutritional indices shows that the “Branca” breed meat is a healthy food that can be ideally incorporated into the human diet at all ages.

Further research should be conducted to define a niche market for “Branca” breed products, and complementary studies aiming to characterize the amino acid and sensorial profiles will lead to the differentiation and valorisation of these products and, consequently, the preservation of the breed.

## Figures and Tables

**Table 1:** Weights and carcass yield of the males and females of the “Branca” breed (mean ± SD, Min and Max).

	Males (n = 20)			Females (n = 20)		
Traits	Mean ± SD	Min	Max	Mean ± SD	Min	Max
LW (g)	3484.00 ^a^ ± 444.10	2815.00	4040.00	2518.50 ^b^ ± 513.90	2060.00	3470.00
CW1 (g)	3188.50 ^a^ ± 417.70	2595.00	3705.00	2334.00 ^b^ ± 496.50	1845.00	3230.00
CW2 (g)	2893.90 ^a^ ± 348.30	2367.00	3437.00	1855.80 ^b^ ± 328.70	1509.00	2.493.00
CW3 (g)	2569.00 ^a^ ± 311.00	2095.00	3050.00	1683.00 ^b^ ± 318.10	1365.00	2325.00
EW (g)	324.90 ^a^ ± 40.60	256.00	387.00	172.80 ^b^ ± 37.30	123.00	232.00
CY1 (%)	91.50 ^a^ ± 0.80	90.40	92.70	92.60 ^a^ ± 1.60	89.60	94.40
CY2 (%)	83.20 ^a^ ± 2.50	78.60	85.60	74.10 ^b^ ± 4.40	66.20	80.30
CY3 (%)	73.80 ^a^ ± 1.90	70.80	75.70	67.10 ^b^ ± 4.60	59.50	73.00
EY (%)	9.40 ^a^ ± 0.70	7.70	10.10	7.00 ^b^ ± 1.50	4.90	9.30

SD—standard deviation; Min—minimum; Max—maximum. LW, live weight at slaughter; CW1, bled and plucked carcass weight; CW2, eviscerated carcass, with head, feet, and edible viscera weight; CW3, eviscerated carcass, without head, feet and edible viscera weight; EW, edible viscera weight (head, feet, gizzard, heart, liver, and kidneys); CY1, bled and plucked carcass yield; CY2, eviscerated carcass yield, with head, feet, and edible viscera; CY3, eviscerated carcass yield, without head, feet and edible viscera; EW, edible viscera yield (head, feet, gizzard, heart, liver, and kidneys); In females, CW2, EW, and their yields do not include the head. Values with different letters within the same row indicate significant differences (*p* ≤ 0.05) between males and females.

**Table 2:** Physicochemical traits of the breast and drumstick meat of males and females of the “Branca” breed (mean ± SD, Min and Max).

		Males			Females		
		Mean ± SD	Min	Max	Mean ± SD	Min	Max
Breast (n = 20)	pH	5.81 ^aA^ ± 0.09	5.69	5.98	5.78 ^aA^ ± 0.02	5.74	5.81
	WHC (%)	55.89 ^aA^ ± 1.79	53.68	58.80	53.13 ^bA^ ± 1.35	50.83	55.32
	PL (%)	12.87 ^aA^ ± 2.17	9.90	15.50	12.07 ^aA^ ± 1.33	10.19	13.84
	Colour						
	L*	50.12 ^aA^ ± 1.87	45.92	52.86	54.68 ^bA^ ± 2.97	49.70	62.78
	a*	4.86 ^aA^ ± 2.01	1.41	8.56	4.82 ^aA^ ± 0.42	4.10	5.89
	b*	10.95 ^aA^ ± 3.22	6.77	16.64	10.05 ^aA^ ± 0.97	6.34	11.68
Drumstick (n = 20)	pH	6.06 ^aB^ ± 0.08	5.98	6.24	6.04 ^aB^ ± 0.05	5.96	6.09
	WHC (%)	56.74 ^aA^ ± 1.95	54.41	59.77	54.02 ^bA^ ± 1.61	52.19	57.22
	PL (%)	10.57 ^aB^ ± 2.35	7.63	13.70	9.41 ^aB^ ± 0.82	8.06	10.54
	Colour						
	L*	40.40 ^aB^ ± 2.93	36.69	47.52	42.02 ^bB^ ± 3.71	28.98	48.41
	a*	16.68 ^aB^ ± 1.00	15.02	18.77	16.08 ^aB^ ± 1.96	13.69	20.60
	b*	10.44 ^aA^ ± 1.65	7.61	12.97	10.09 ^aA^ ± 1.43	7.16	14.21

SD—standard deviation; Min—minimum; Max—maximum. WHC—water-holding capacity; PL—pressing loss; L*—lightness; a*—redness; b*—yellowness. Values with different small letters within the same row indicate significantly differences (*p* ≤ 0.05) between males and females. Means with different capital letters in the same column indicate significantly differences (*p* ≤ 0.05) between breast and drumstick.

**Table 3:** Chemical composition of the breast and drumstick meat of males and females of the “Branca” breed (mean ± SD, Min and Max) (results expressed in % of fresh weight).

		Males			Females		
		Mean ± SD	Min	Max	Mean ± SD	Min	Max
Breast (n = 20)	Moisture (%)	73.40 ^aA^ ± 0.77	72.21	75.20	73.21 ^aA^ ± 0.47	72.25	74.07
	Ash (%)	1.21 ^aA^ ± 0.06	1.04	1.31	1.17 ^bA^ ± 0.04	1.09	1.27
	Protein (%)	24.13 ^aA^ ± 0.68	22.45	25.29	23.98 ^aA^ ± 0.44	22.79	24.68
	Lipids (%)	0.22 ^aA^ ± 0.15	0.04	0.56	0.41 ^bA^ ± 0.15	0.10	0.67
Drumstick (n = 20)	Moisture (%)	74.22 ^aB^ ± 1.12	72.58	76.56	71.54 ^bB^ ± 1.30	69.27	73.11
	Ash (%)	1.15 ^aB^ ± 0.06	1.02	1.23	1.09 ^bB^ ± 0.03	1.03	1.13
	Protein (%)	20.12 ^aB^ ± 0.53	19.15	20.95	20.17 ^aB^± 0.57	19.44	21.31
	Lipids (%)	1.03 ^aB^ ± 0.47	0.19	1.87	2.81 ^bB^ ± 0.92	1.72	4.40

SD—standard deviation; Min—minimum; Max—maximum. Values with different small letters within the same row indicate significantly differences (*p* ≤ 0.05) between males and females. Means with different capital numbers in the same column indicate significantly differences (*p* ≤ 0.05) between breast and drumstick.

**Table 4:** Fatty acid profile of the breast and drumstick meat of males and females of the “Branca” breed (mean ± SD, Min and Max) (results expressed as % of total fatty acids).

		Males			Females		
	Fatty Acid (%)	Mean ± SD	Min	Max	Mean ± SD	Min	Max
Breast (n = 20)	C14:0	0.47 ^aA^ ± 0.08	0.32	0.59	0.65 ^bA^ ± 0.09	0.52	0.80
	C16:0	19.83 ^aA^ ± 1.96	15.81	23.12	19.79 ^aA^ ± 1.70	17.95	23.18
	C18:0	9.21 ^aA^ ± 0.69	7.61	10.05	7.54 ^bA^ ± 1.01	6.10	9.62
	Others	1.56 ^aA^ ± 0.35	1.12	2.37	1.17 ^bA^ ± 0.28	0.87	1.76
	⅀SFA	**31.07** ^aA^ **± 2.05**	**26.91**	**33.88**	**29.14** ^bA^ **± 2.22**	**26.05**	**32.95**
	C16:1	2.54 ^aA^ ± 0.63	1.26	3.51	2.86 ^aA^ ± 0.75	1.99	4.40
	C18:1	34.19 ^aA^ ± 2.18	30.75	37.22	38.27 ^bA^ ± 2.57	33.92	42.36
	C20:1	0.31 ^aA^ ± 0.04	0.26	0.40	0.28 ^aA^ ± 0.04	0.21	0.36
	Others	1.49 ^aA^ ± 0.41	0.86	2.34	1.14 ^bA^ ± 0.37	0.66	1.98
	⅀MUFA	**38.53** ^aA^ **± 2.10**	**35.07**	**41.22**	**42.55** ^bA^ **± 2.93**	**38.15**	**47.67**
	C18:2n-6	17.05 ^aA^ ± 3.30	11.99	24.36	19.63 ^bA^ ± 3.83	14.59	25.67
	C20:3n-6	0.28 ^aA^ ± 0.06	0.15	038	0.19 ^bA^ ± 0.06	0.12	0.28
	C20:4n-6	6.24 ^aA^ ± 1.74	3.43	8.52	4.06 ^bA^ ± 1.56	2.31	7.07
	⅀n-6-PUFA	23.67 ^aA^ ± 2.02	20.81	28.06	24.02 ^aA^ ± 3.43	19.51	29.23
	C18:3n-3	0.53 ^aA^ ± 0.15	0.31	0.79	0.54 ^aA^ ± 0.15	0.30	0.82
	C22:5n-3	0.81 ^aA^ ± 0.31	0.37	1.30	0.29 ^bA^ ± 0.19	0.11	0.64
	C22:6n-3	0.73 ^aA^ ± 0.21	0.43	1.13	0.82 ^aA^ ± 0.28	0.40	1.47
	⅀n-3-PUFA	2.03 ^aA^ ± 0.48	1.34	2.82	1.62 ^bA^ ± 0.39	1.07	2.46
	⅀LC-PUFAS	8.36 ^aA^ ± 2.16	4.86	11.12	5.57 ^bA^ ± 1.95	3.17	9.45
	⅀PUFA	**26.08** ^aA^ **± 2.03**	**23.16**	**30.20**	**25.94** ^aA^ **± 3.51**	**21.62**	**30.88**
	TRANS	0.25 ^aA^ ± 0.07	0.17	0.44	0.23 ^aA^ ± 0.03	0.17	0.28
	EFA	23.82 ^aA^ ± 2.16	20.86	28.60	24.23 ^aA^ ± 3.50	19.63	29.49
	PUFA/SFA	0.85 ^aA^ ± 0.13	0.69	1.06	0.90 ^aA^ ± 0.17	0.66	1.19
	n-6/n-3	12.37 ^aA^ ± 3.44	8.23	18.00	14.36 ^aA^ ± 3.36	9.44	19.80
Drumstick (n = 20)	C14:0	0.58 ^aB^ ± 0.09	0.42	0.73	0.73 ^bB^ ± 0.13	0.55	0.93
	C16:0	19.86 ^aA^ ± 2.08	16.92	23.34	18.64 ^bB^ ± 1.50	16.89	21.44
	C18:0	9.83 ^aB^ ± 0.85	8.56	11.24	7.54 ^bA^ ± 0.79	6.43	9.46
	Others	1.22 ^aB^ ± 0.18	0.96	1.62	1.15 ^aA^ ± 0.18	0.95	1.52
	⅀SFA	**31.50** ^aA^ **± 1.79**	**28.58**	**33.62**	**28.05** ^bB^ **± 1.80**	**25.29**	**31.05**
	C16:1	3.73 ^aB^ ± 0.87	2.35	4.92	4.15 ^aB^ ± 0.95	2.98	5.82
	C18:1	34.71 ^aA^ ± 1.34	31.94	36.74	38.78 ^bA^ ± 1.68	35.32	41.35
	C20:1	0.28 ^aA^ ± 0.12	0.06	0.41	0.29 ^aA^ ± 0.03	0.24	0.35
	Others	0.95 ^aB^ ± 0.11	0.78	1.20	0.91 ^aB^ ± 0.13	0.71	1.15
	⅀MUFA	**39.67** ^aA^ **± 1.84**	**35.69**	**41.77**	**44.13** ^bA^ **± 2.18**	**40.12**	**47.47**
	C18:2n-6	20.80 ^aB^ ± 2.38	16.82	24.69	21.33 ^aA^ ± 3.75	16.94	27.93
	C20:3n-6	0.20 ^aB^ ± 0.04	0.12	0.31	0.15 ^bB^ ± 0.04	0.10	0.23
	C20:4n-6	3.80 ^aB^ ± 0.75	2.42	4.74	2.50 ^bB^ ± 0.40	1.96	3.36
	⅀n-6-PUFA	24.93 ^aA^ ± 2.23	21.83	28.85	24.12 ^aA^ ± 3.45	19.93	30.48
	C18:3n-3	0.66 ^aB^ ± 0.14	0.37	0.86	0.60 ^aA^ ± 0.16	0.36	0.93
	C22:5n-3	0.36 ^aB^ ± 0.12	0.18	0.53	0.15 ^bB^ ± 0.09	0.06	0.37
	C22:6n-3	0.36 ^aB^ ± 0.10	0.16	0.53	0.45 ^bB^ ± 0.07	0.32	0.55
	⅀n-3-PUFA	1.35 ^aB^ ± 0.24	0.89	1.63	1.17 ^bB^ ± 0.24	0.83	1.59
	⅀LC-PUFAS	5.05 ^aB^ ± 0.81	3.34	6.09	3.51 ^bB^ ± 0.50	2.81	4.64
	⅀PUFA	**26.70** ^aA^ **± 2.37**	**23.56**	**30.92**	**25.66** ^aA^ **± 3.51**	**21.54**	**32.48**
	TRANS	0.28 ^aA^ ± 0.06	0.19	0.40	0.29 ^aB^ ± 0.05	0.23	0.43
	EFA	25.26 ^aB^ ± 2.27	22.13	29.26	24.43 ^aA^ ± 3.53	20.32	31.10
	PUFA/SFA	0.85 ^aA^ ± 0.11	0.70	1.02	0.93 ^aA^ ± 0.19	0.69	1.28
	n-6/n-3	19.15 ^aB^ ± 3.97	13.43	26.31	20.93 ^aB^ ± 4.26	13.47	27.55

SD—standard deviation; Min—minimum; Max—maximum.SFA—saturated fatty acids; MUFA—monounsaturated fatty acids; PUFA—polyunsaturated fatty acids; EFA—essential fatty acids (including linoleic acid, linolenic acid, and arachidonic acid); PUFA/SFA—ratio between polyunsaturated and saturated fatty acids; n-6/n-3—ratio between the sum of n-6 and n-3 fatty acids; Values with different small letters within the same row indicate significantly differences (*p* ≤ 0.05) between males and females. Means with different capital numbers in the same column indicate significantly differences (*p* ≤ 0.05) between breast and drumstick. The bold is the ⅀ of different types of fatty acids.

**Table 5:** Mineral composition of the breast and drumstick meat from the males and females of the “Branca” breed (mean ± SD, Min and Max) (results expressed in mg/100 g of dry weight).

		Males			Females		
	Minerals	Mean ± SD	Min	Max	Mean ± SD	Min	Max
Breast (n = 20)	Macronutrients (mg/100 g)						
	Phosphorous	730.33 ^aA^ ± 40.98	611.97	790.11	705.32 ^aA^ ± 46.01	621.83	791.54
	Potassium	1828.62 ^aA^ ± 81.74	1694.15	1944.03	1722.59 ^bA^ ± 70.82	1565.17	1808.46
	Calcium	45.74 ^aA^ ± 9.71	28.77	61.90	57.71 ^bA^ ± 10.85	38.81	75.13
	Magnesium	125.86 ^aA^ ± 3.17	119.27	131.77	114.09 ^bA^ ± 9.03	99.00	127.00
	Sodium	559.43 ^aA^ ± 41.99	489.05	654.95	498.01 ^bA^ ± 68.81	395.00	639.70
	Trace elements (mg/100 g)						
	Iron	3.67 ^aA^ ± 0.81	2.63	5.22	6.12 ^bA^ ± 0.59	5.08	7.01
	Zinc	5.75 ^aA^ ± 0.95	4.28	7.55	4.01 ^bA^ ± 0.59	3.08	5.38
	Manganese	0.31 ^aA^ ± 0.09	0.17	0.47	1.21 ^bA^ ± 0.45	0.59	1.85
	Copper	0.41 ^aA^ ± 0.05	0.30	0.47	0.51 ^bA^ ± 0.09	0.35	0.70
Drumstick (n = 20)	Macronutrients (mg/100 g)						
	Phosphorous	660.60 ^aB^ ± 39.45	604.12	742.16	531.49 ^bB^ ± 65.51	400.41	642.19
	Potassium	1714.09 ^aB^ ± 94.80	1566.42	1868.56	1513.83 ^bB^ ± 117.00	1281.84	1721.25
	Calcium	48.28 ^aA^ ± 9.90	31.02	58.47	68.09 ^bB^ ± 14.32	48.46	96.36
	Magnesium	109.19 ^aB^ ± 4.18	103.19	117.82	87.38 ^bB^ ± 10.64	69.40	100.75
	Sodium	734.22 ^aB^ ± 53.40	638.67	822.75	566.39 ^bB^ ± 53.78	446.75	646.50
	Trace elements (mg/100 g)						
	Iron	6.93 ^aB^ ± 1.11	5.71	9.33	9.86 ^bB^ ± 1.10	8.53	11.46
	Zinc	16.45 ^aB^ ± 1.17	14.61	18.27	10.84 ^bB^ ± 1.59	7.49	13.02
	Manganese	0.38 ^aB^ ± 0.08	0.25	0.50	1.15 ^bA^ ± 0.40	0.50	1.83
	Copper	0.55 ^aB^ ± 0.06	0.42	0.69	0.67 ^bB^ ± 0.15	0.40	1.04

SD—standard deviation; Min—minimum; Max—maximum. Values with different small letters within the same row indicate significantly differences (*p* ≤ 0.05) between males and females. Means with different capital numbers in the same column indicate significantly differences (*p* ≤ 0.05) between breast and drumstick.

## Data Availability

The raw data have been submitted to CISAS-IPVC (Center for Research and Development in Agrifood Systems and Sustainability—Polytechnic Institute of Viana do Castelo) and are available on request.
